# Geospatial variation and associated factors of unintended pregnancy among women of reproductive age in Ethiopia: Geographically Weighted Regression Analysis

**DOI:** 10.1371/journal.pone.0337282

**Published:** 2026-02-23

**Authors:** Tesfaye Deribe Bedada, Bereket Yilma Beshah, Hailemariam Kassahun Desalegn, Biruktawit lelisa Eticha, Tamir Wondim Desta, Andualem Addisu Birlie

**Affiliations:** 1 Department of Health Informatics, School of Public Health, Institute of Health, Bule Hora University, Bule Hora, Ethiopia; 2 Department of Health Informatics, School of Public Health, College of Medicine and Health Sciences, Wollo University, Dessie, Ethiopia; 3 Department of Health Informatics, School of Public Health, College of Medicine and Health Sciences, Wachemo University, Hosanna, Ethiopia; 4 Department of Health Informatics, Arba Minch Health Science College, Arba Minch, Ethiopia; 5 Department of Health Informatics, College of Medicine and Health Sciences, Samara University, Samara, Ethiopia; Universidad Peruana Cayetano Heredia Facultad de Medicina, PERU

## Abstract

**Background:**

Unintended pregnancies are a serious public health concern that has several effects on the health of mothers and children. However, no studies have been conducted on unintended pregnancy using geographically weighted regression in the 2016 Ethiopian demographic and health survey. Therefore, this study assessed the geospatial and associated factors of unintended pregnancy in Ethiopia using a 2016 demographic and health survey.

**Methods:**

A cross-sectional study used data from the 2016 demographic and health survey and included 7589 women of the reproductive age. Spatial analysis and mapping were conducted using ArcGIS version 10.8. Spatial clusters were identified using the Bernoulli model in SaTScan 10.1. Geographically weighted regression was used to assess associated factors, with significance at p < 0.05.

**Results:**

Unintended pregnancy showed a clustered spatial pattern. SaTScan found 171 primary significant clusters (risk ratio = 1.86, p < 0.001) in Addis Ababa, Oromia, and SNNPR. A geographically weighted regression revealed that maternal age (35–49 years), maternal primary education, high socio economic status, and distance to a health facility (a big problem) were significant factors contributing to unintended pregnancy.

**Conclusions:**

Hotspot analysis identified statistically significant hotspot areas of unintended pregnancy in Amhara, Addis Ababa, Oromia, and SNNPR. Statistically significant coldspot areas of unintended pregnancy were observed in Afar, Dire Dawa, and Somali. Maternal age (35–49 years), maternal primary education, high socio economic status, and distance to health facility (big problem) were statistically significant factors of unintended pregnancy. These results show that the capital city and some key rural and ethnic areas had higher rates of unintended pregnancies, especially among relatively older women with high socioeconomic status and basic education, which indicates that sexual and reproductive health education needs to be strengthened and carried out at all levels, including among high socioeconomic groups.

## Background

Unintended pregnancy is a pregnancy that is either unwanted or mistimed [1]. Unintended pregnancies can happen for many reasons, including non-use of family planning and contraceptive methods, having unexpected sexual encounters like rape, and lack of awareness about family planning services, low understanding of the reproductive cycle, no education or awareness of refusing sex when not protected [2–4]. Women with unintended pregnancies are more likely to have preterm delivery and other adverse pregnancy outcomes [5]. Unintended pregnancy continues to present a neglected global public health burden. Worldwide, from 2015 to 2019, there were an estimated 121 million unintended pregnancies occurring each year [2]. The estimated proportion of all pregnancies that were unintended was 29% in Sub-Saharan Africa, varying between 10.8% in Nigeria, and 54.5% in Namibia [6]. Unintended pregnancies occur in high-income countries at a rate of 66 per 1,000 women, and in low-and middle income countries, the rate increases to 93 per 1,000 women [1]. Unintended pregnancies have become more common in countries with lower and middle-incomes countries, which often lead to serious challenges for both mothers and their children [2]. These can include a weaker bond between mother and child, a higher chance of facing physical abuse, difficulties in relationships with partners, unsafe abortions, suffering from depression, causing family stress, having fewer job opportunities, and struggling with education, particularly for those living in rural areas [2,7–9].

According to previous study reports, several factors are associated with unintended pregnancy. These include maternal age [10], maternal educational level [11], maternal marital status [12], distance from the nearest health facility [13], parity [14], wealth index [15], knowledge of ovulation cycle [16], and working status [15]. The Sustainable Development Goals (SDGs) set out targets to make all women and couples have the right to freely decide how many children they want and when to have them, with the global targets that by 2030, all births are wanted and every child is valued [17]. To make this happen, strong efforts are crucial to cut down on unintended pregnancies; this can help prevent unsafe abortions, reduce the number of mothers and children who die, and ease the financial and social strain on families and communities [18]. The majority of earlier research employed global models, which produce generalized findings and limit their ability to capture local variations. By using Geographically Weighted Regression (GWR) on 2016 EDHS data, this study fills that gap by enabling a localized analysis of the geospatial and associated factors of unintended pregnancy.

## Methods and materials

### Study design, period and area

**Fig 1:**
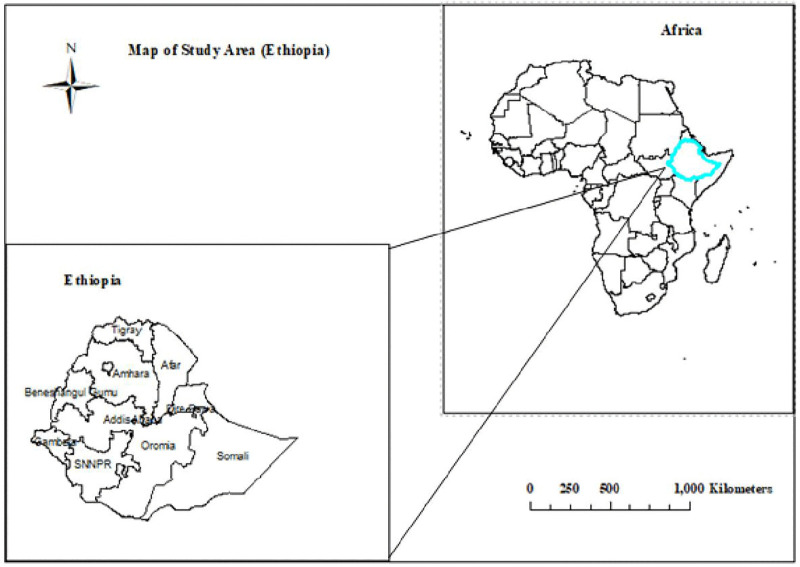
Map of study area (Ethiopia). **Map data source:** **https://africaopendata.org/dataset/ethiopiashapefiles**.

In 2016 Ethiopia Demographic and Health Survey (EDHS), the Central Statistical Agency (CSA) carried out a community-based cross-sectional study in Ethiopia between January 18 and June 27, 2016 [19]. There are two city-administrative states (Addis Ababa and Dire-Dawa) and nine regional states (Afar, Amhara, Benishangul-Gumuz, Gambella, Harari, Oromia, Somali, Southern Nations, Nationalities, and People’s Region (SNNP), and Tigray) (**Fig 1).**

### Source and study population

All women of reproductive ages (15–49 years) in the EDHS 2016 were the source and study population. All women who have complete data on the status of pregnancy wantedness in EDHS 2016 were included in the study. However, all women observed in enumeration areas with zero coordinates on the status of pregnancy wantedness were excluded from the study.

### Sampling procedures

The DHS provided secondary data for this study using a stratified multistage sampling technique. In the initial phase, urban and rural areas were considered distinct strata, and enumeration areas were chosen using a probability proportional to size. In the second phase, a systematic sampling method was used to select households within each cluster. Lastly, structured questionnaires were used to interview consenting eligible women [20].

### Study variables

The dependent variable in this study was unintended pregnancy [1]. The independent variables considered were maternal age [10], maternal educational level, husband educational level [11], maternal marital status [12], distance from the nearest health facility [13], parity [14], wealth index [15], knowledge of ovulation cycle [16], working status, and husband working status [15].

### Operational definition

#### Unintended pregnancy.

Unintended pregnancy is considered an outcome variable, with two possible outcomes (yes or no). All women of reproductive age who participated in the 2016 EDHS were asked if they were pregnant at the time of the survey. The woman was asked if she wanted the pregnancy at the time, later, or not at all. The outcome variable was deemed “no” when the woman stated that she wanted the pregnancy at the time of the survey, indicating that the pregnancy was intended. Conversely, the outcome variable was deemed unintended and coded “yes” if the woman stated that the pregnancy was not wanted at all or was wanted later.

#### Knowledge of the fertility period.

Knowledge of the fertility period was assessed by the standardized question, “When do you think a woman’s fertility period is?” Respondents that answered “In the middle of the menstrual cycle” were classified as having good knowledge about the fertility period, whereas “When do you think a woman’s fertility period is?” Respondents that answered “During her period,” “After her period finished,” “Before her period started,” or “At any time of a month” were categorized as having poor knowledge about fertility periods [21–23].

### Data quality management

Robust procedures were put in place to guarantee the quality of the data, including ongoing field supervision and thorough training for supervisors, field editors, and data collectors. To take into account the linguistic preferences of the participants, standardized questionnaires were translated into both national and local languages; data entry and management were supervised by data processing specialists. In order to address missing values and guarantee analytical consistency, post-collection procedures entailed combining the datasets of men and women and using systematic recoding and reclassification [24].

### Data processing

This study made use of nationally representative cross-sectional DHS data from Ethiopia that was made publicly available and taken from individual recode (IR) files. Microsoft Excel 2019 and STATA version 17 were used for data management, which included handling missing values, cleaning, recoding, and appending data. The [IW = wgt] command in STATA was used to apply sampling weights (v005) in order to guarantee representativeness and produce accurate estimates. ArcGIS 10.8 and SaTScan version 10.1 were used for the spatial analyses.

#### Spatial autocorrelation and hotspot analysis.

Using Global Moran’s I to measure spatial autocorrelation; the study evaluated the spatial variation of unintended pregnancies in Ethiopia. Moran’s I values range from −1 (indicating dispersion) to +1 (indicating clustering), with values near zero suggesting a random spatial pattern [25]. The Getis-Ord Gi* statistic was used in hotspot analysis to find significant spatial clusters. Hotspots (high-value clusters) were defined as clusters with Z-scores > 1.96 and p-values < 0.05, whereas coldspots (low-value clusters) were defined as clusters with Z-scores < −1.96 and p-values < 0.05 [26]. A p-value ≥ 0.05 would indicate that the cluster is not statistically significant and should not be classified as a hotspot or coldspot [27].

#### Spatial interpolation.

Spatial interpolation was employed to estimate the unintended pregnancy in unsampled areas based on data from sampled clusters [28,29]. The study applied both deterministic and geostatistical interpolation methods and selected ordinary kriging geostatistical interpolation methods as the most accurate technique due to its lowest mean predicted error (MPE) of 0.001 and root mean square predicted error (RMSPE) of 0.1573 (**Table 2).**

**Table 1:** Descriptive statistics for each selected variable in Ethiopia.

		Pregnancy	
Characteristics	Category	Frequency	Percent
Maternal Age	15–19	339	4.50
	20–34	5,292	69.70
	35–49	1,959	25.80
Marital Status	Single	55	0.70
	Married	7,109	93.70
	Widowed	96	1.30
	Divorced	330	4.30
Maternal educational level	No education	4,791	63.20
	Primary	2,150	28.30
	Secondary	420	5.50
	Higher	229	3.0
Husband educational level	No education	3,345	44.10
	Primary	2,732	36.0
	Secondary	613	8.10
	Higher	376	5.0
Wealth index	Poor	3,285	43.30
	Middle	1,580	20.80
	Rich	2,682	35.30
Maternal Working status	Not working	5,384	71.0
	Working	2,162	28.50
Husband working status	Not employed	524	6.90
	Employed	6,266	82.60
Parity	0-1	1,351	17.80
	2-4	5,689	75.0
	5+	231	3.0
Knowledge of the ovulation cycle	Poor knowledge	1,175	15.50
	Good knowledge	6,096	80.30
Distance to the nearest health facility	Big problem	4,259	56.10
	Not a big problem	3,011	39.70

**Table 2:** Interpolation method comparison of unintended pregnancy.

	Parameter	
Interpolation method	Mean predicted error (MPE)	Root mean square predicted error (RMSPE)
Deterministic interpolation		
Inverse distance weighted	0.003	0.1698
Geostatistical interpolation		
**Ordinary kriging**	**0.001**	**0.1573**
Universal kriging	0.002	0.1674
Simple kriging	0.003	0.1739
Disjunctive kriging	0.004	0.1740
Indictor kriging	0.007	0.4609
Probability kriging	0.007	0.4609

#### Spatial scan statistics.

Kuldorff’s Bernoulli-based spatial scan statistics in SaTScan version 10.1 were used for purely spatial analysis in order to identify statistically significant clusters of unintended pregnancy [28,29]. Unintended pregnancy women were categorized as cases, and intended pregnancy women as controls. The maximum size of a spatial cluster was set at ≤25% of the total population. Using p-values derived from 999 Monte Carlo replications and likelihood ratio tests, the statistical significance of the clusters was evaluated. The non-overlapping option was used to find secondary clusters, while the primary cluster was determined to be the most likely cluster within the scanning window. ArcGIS version 10.8 was used to map every cluster that was found.

### Ethical approval and consent to participate

The data are secondary and publicly available. All DHS datasets are anonymzed and de-identified before release. Permission for data access was acquired from the DHS through an online request accompanied by a written letter detailing the study’s objectives, and permission was finally obtained from the DHS program: http://www.dhprogram.com.

#### Spatial regression.

To determine if the data satisfied the assumptions of Ordinary Least Squares (OLS), exploratory regression analysis was performed. Stationarity (Koenker statistic p > 0.05), spatial independence of residuals, normality (Jarque-Bera statistic p > 0.05), lack of multicollinearity (variance inflation factor < 7.5), factors statistical significance (p < 0.05), and model performance (high adjusted R-square and low Akaike Information Criterion) were among these [30–32]. Moreover, OLS was used to select the best variables for further spatial analysis in unintended pregnancy.

#### Geographically Weighted Regression (GWR).

GWR models spatial non-stationarity by allowing regression coefficients to vary by spatial units, estimating the best bandwidth for all factors. With this statistics a positive coefficient indicates that the factors and unintended pregnancy move in the same direction, and a negative coefficient indicates the opposite direction [30,31]. Spatial heterogeneity for each coefficient was explored based on the corrected Akaike Information Criterion (AICc) and adjusted R-squared values produced by the global OLS and local GWR models. The model with the lowest Akaike Information Criterion (AICc) and highest adjusted R-squared is the best-fit model for estimating local parameters [25]. In order to ensure optimal local model performance and account for spatially variable data density, an adaptive bandwidth was employed based on the minimization of the corrected Akaike Information Criterion (AICc).

## Results

### Descriptive statistics

Out of 7,589 reproductive-age women, the actual prevalence of unintended pregnancy was 5,489 (72.3%). The descriptive characteristics of the study variables are summarized in Table 1.

#### Spatial autocorrelation (Global Moran’s Index) analysis.

**Fig 2:**
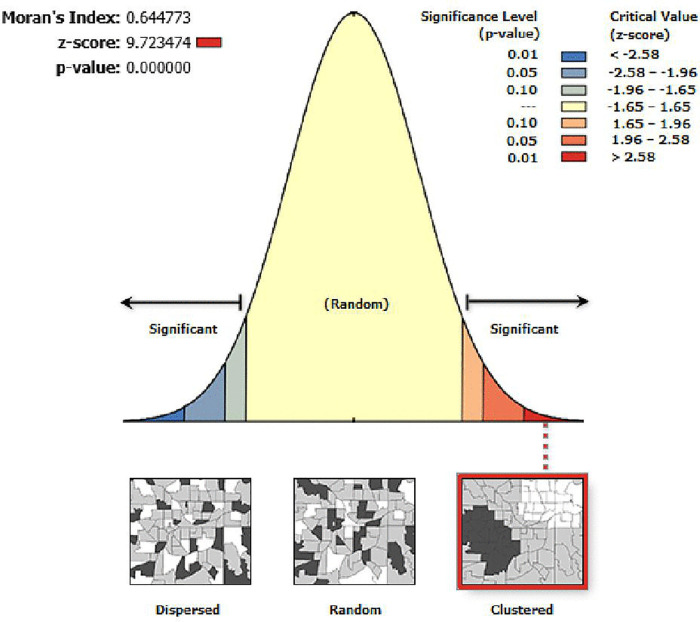
Spatial autocorrelation of unintended pregnancy in Ethiopia.

Spatial autocorrelation revealed a high and statistically significant clustering pattern of unintended pregnancy, with Moran’s Index of 0.64 (p-value less than 0.001) and Z-score of 9.72, indicating there was less than 1% likelihood that this clustered pattern could be the result of random choice (Fig 2). This indicates that unintended pregnancy is not randomly distributed across the Ethiopia regions. This means that unintended pregnancies are clustered in geographic locations, forming areas with high prevalence (hotspots) and others with low prevalence (coldspots) rather than being randomly distributed.

#### Hotspot analysis (Getis-Ord Gi*).

**Fig 3:**
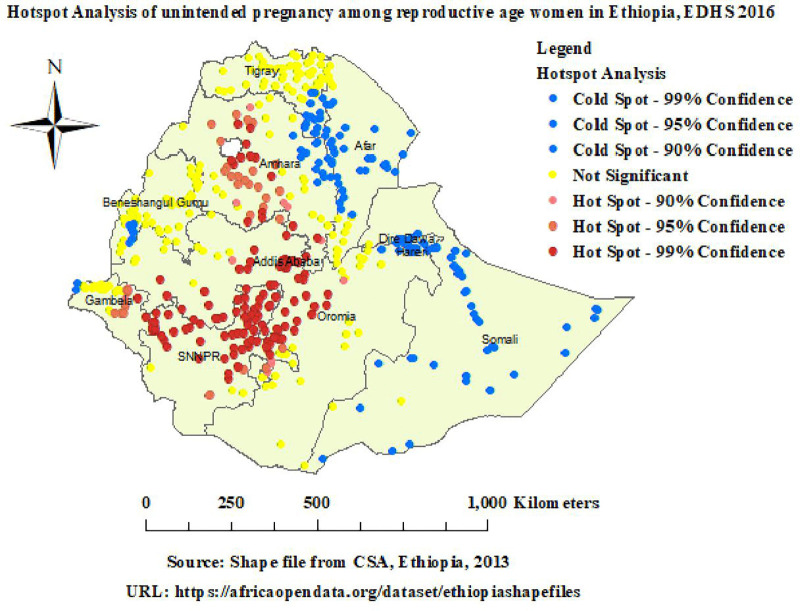
Hotspot analysis of unintended pregnancy in Ethiopia. Map data source: https://africaopendata.org/dataset/ethiopiashapefiles.

Red-colored areas represent statistically significant hotspots; Hotspot analysis identified statistically significant hotspot areas of unintended pregnancy in Amhara, Addis Ababa, Oromia, and SNNPR. Blue-colored areas denote significant coldspots; statistically significant coldspot areas of unintended pregnancy were observed in Afar, Dire Dawa, and Somali (Fig 3).

#### Spatial interpolation.

**Fig 4:**
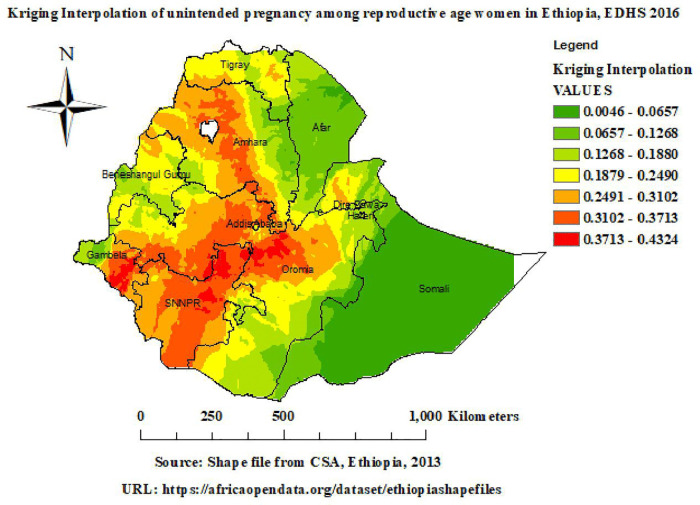
Interpolation of unintended pregnancy. Map data source: https://africaopendata.org/dataset/ethiopiashapefiles.

In this study, ordinary kriging geostatistical interpolation methods were applied for predicting unintended pregnancy in unobserved areas (**Table 2).** As shown in Fig 4 below, ordinary kriging interpolation analysis predicted unintended pregnancy increases from green to red-colored areas in EDHS 2016. Red areas indicated areas with high predictions of unintended pregnancy in Amhara, Addis Ababa, Oromia, and SNNPR. Green areas indicate areas with low predictions in Afar, Dire Dawa, and Somali.

#### Spatial SaTScan.

**Fig 5:**
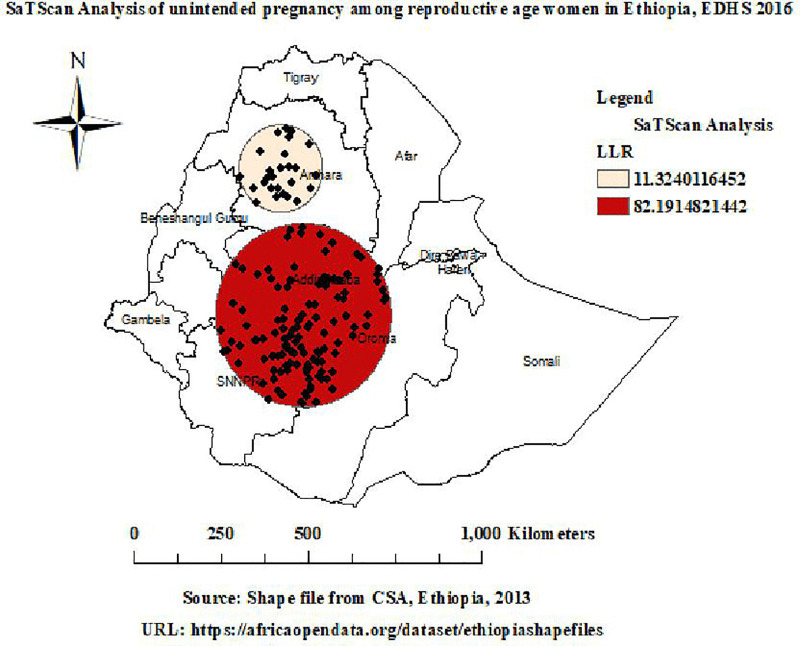
Spatial SaTScan analysis of unintended pregnancy. Map data source: https://africaopendata.org/dataset/ethiopiashapefiles.

Out of a total of 622 clusters, 197 were statistically significant clusters of unintended pregnancy, which were categorized into one primary and one secondary cluster. The primary clusters were 171 statistically significant clusters observed in Addis Ababa, Oromia, and SNNPR and centered at 8.15920N, 38.1893E, 253.17 km with an LLR of 82.19, RR: 1.86, at p-value less than 0.001. It indicated that women inside the spatial window had an 86% higher risk of unintended pregnancy than women outside the window. The secondary clusters were 26 statistically significant clusters and observed in Amhara region and centered at 11.7930N, 37.5846E, 120.71 km with an LLR of 11.32, RR: 1.60, at p-value less than 0.001. It indicated that women inside the spatial window had a 60% higher risk of unintended pregnancy than women outside the window (**Table 3 and Fig 5**).

**Table 3:** Significant clusters of unintended pregnancy.

Cluster	#location	Number of Population	#Case	RR	LLR	Coordinates/Radius	P-value
Primary	171	1796	558	1.86	82.19	8.15920N,38.1893E,253.17 km	<0.001
Secondary	26	297	94	1.60	11.32	11.7930N,37.5846E,120.71 km	<0.001

#### Spatial regression analysis.

Initially, all candidate variables were included in an exploratory regression analysis to find factors that were significantly associated with unintended pregnancy. Based on this analysis, maternal age, maternal education, wealth index, and the distance to the health facility were chosen for further analysis. GWR was then applied to determine these key factors and unintended pregnancy across regions of Ethiopia.

As shown in **Table 4** below, the coefficients were all statistically significant at a p-value less than 0.005; multicollinearity did not exist among independent variables, as all variance inflation factors (VIF) were below 7.5. OLS revealed explanatory power with an R-squared of 23%, an adjusted R-squared of 21%, and Akaike’s information criteria (AICc) of (−444.21). The Joint Wald statistic was also significant (p-value less than 0.001), indicating the overall model was statistically significant. The significant Koenker (BP) value (23.01, p-value less than 0.001) implies that the associations between the independent variable and dependent variable were spatially non-stationary (heteroscedasticity), which implies variation of coefficient across clusters. Therefore, the significant Koenker test supports the use of GWR regression model. The results of the GWR model were summarized in **Table 5**.

**Table 4:** Global beta coefficients of the OLS model summary and diagnostics.

Variable	Coefficient	Std Error	Probability	Robust probability	VIF
Intercept	+0.0723	0.0251	<0.001	<0.001	
Maternal age (35–49 years)	+0.1088	0.0412	<0.001	<0.001	1.05
Maternal primary education	+0.1263	0.0328	<0.001	<0.001	1.14
high socioeconomic status	+0.0896	0.0226	<0.001	<0.001	1.87
Distance to HF (a big problem)	+0.0638	0.0256	<0.001	<0.001	1.68
OLS regression diagnostics					
Diagnostic criteria	Magnitude	P-value			
Akaike’s information criteria (AICc)	−444.21				
R-squared	0.23				
Adjusted R-squared	0.21				
Joint F-statistic	11.21	<0.001			
Joint Wald statistic	42.20	<0.001			
Koenker (BP) statistic	23.01	<0.001			
Jarque-Bera statistic	47.8	<0.001			

**Table 5:** GWR model for unintended pregnancy.

Independent Variable	Maternal age (35–49 years), maternal primary education, high socioeconomic status, Distance to a health facility (a big problem) is positively associated with unintended pregnancy.
Residual Squares	9.03
Effective number	178.82
Sigma	0.15
Akaike’s information criteria (AICc)	−493.6
R-squared	0.584
Adjusted R-squared	0.527

For model comparison, the corrected Akaike Information Criteria (AICc) and adjusted R-squared were used. OLS revealed an R-squared of 23%, an Adjusted R-squared of 21%, and Akaike’s information criteria (AICc) of (−444.21), and GWR significantly improved the models explanatory power, raising an R-squared to 58.4%, an adjusted R-squared to 52.7%, and lowering Akaike’s information criteria (AICc) to (−493.6) (**Table 6),** and finally, GWR was applied to determine the local relationship between maternal age (35–49 years), maternal primary education, the high socioeconomic status, distance to health facility (a big problem) and unintended pregnancy in Ethiopia.

**Table 6:** Model comparisons between OLS and GWR for unintended pregnancy.

Model comparison		
Parameter	OLS model	GWR model
R-squared	23%	58.4%
Adjusted R-squared	21%	52.7%
Akaike’s information criteria (AICc)	−444.21	−493.6

**Fig 6:**
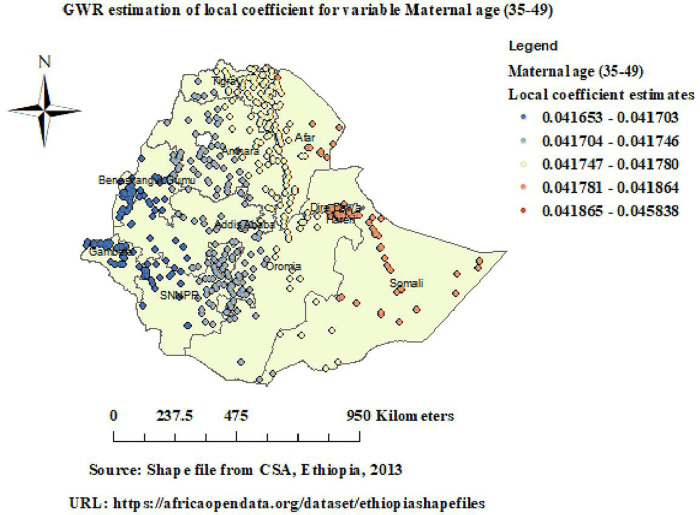
GWR of maternal age (35-49 years) in unintended pregnancy. Map data source: https://africaopendata.org/dataset/ethiopiashapefiles.

GWR analysis revealed that the relationship between maternal age (35-49 years) and unintended pregnancy varies across Ethiopia, with positive coefficients varying from (0.041) to (0.046). Strong positive coefficients varying from (0.042) to (0.046) and were observed in Eastern Afar, Dire Dawa, and Somali (Fig 6).

**Fig 7:**
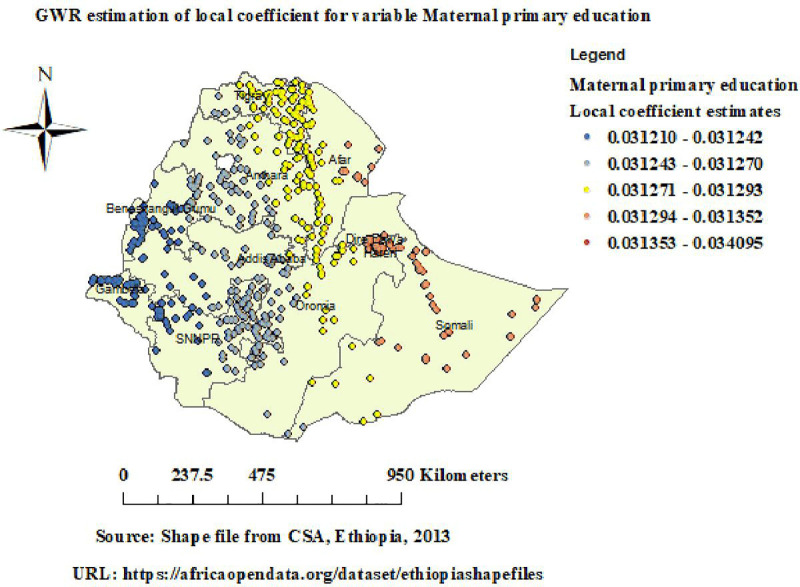
GWR of maternal primary education in unintended pregnancy. Map data source: https://africaopendata.org/dataset/ethiopiashapefiles.

GWR analysis revealed that the relationship between maternal primary education and unintended pregnancy varies across Ethiopia, with positive coefficients varying from (0.03) to (0.034). Strong positive coefficients varying from (0.031) to (0.034) and were observed in Eastern Afar, Dire Dawa, and Somali (Fig 7).

**Fig 8:**
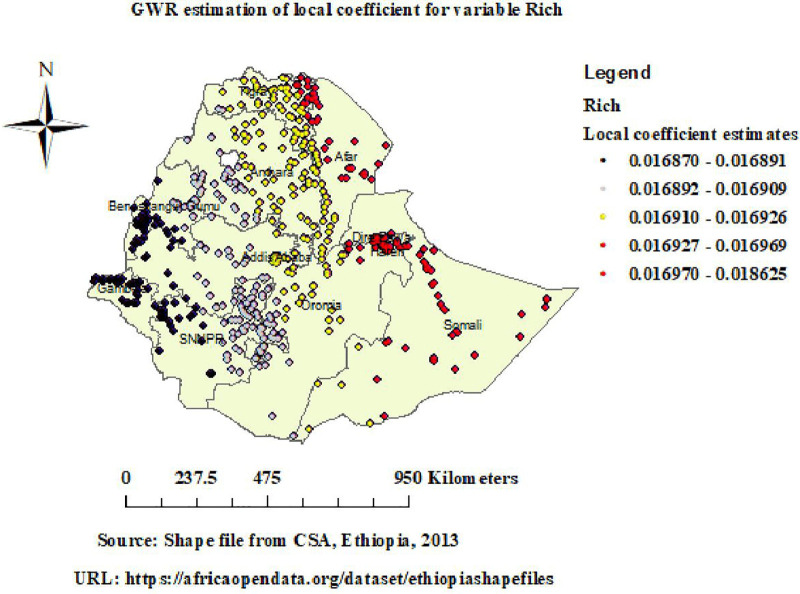
GWR of high socioeconomic status in unintended pregnancy. Map data source: https://africaopendata.org/dataset/ethiopiashapefiles.

GWR analysis revealed that the relationship between high socioeconomic status and unintended pregnancy varies across Ethiopia, with positive coefficients varying from (0.012) to (0.019). Strong positive coefficients varying from (0.017) to (0.019) and were observed in Eastern Tigray, Afar, Dire Dawa, and Somali (Fig 8).

**Fig 9:**
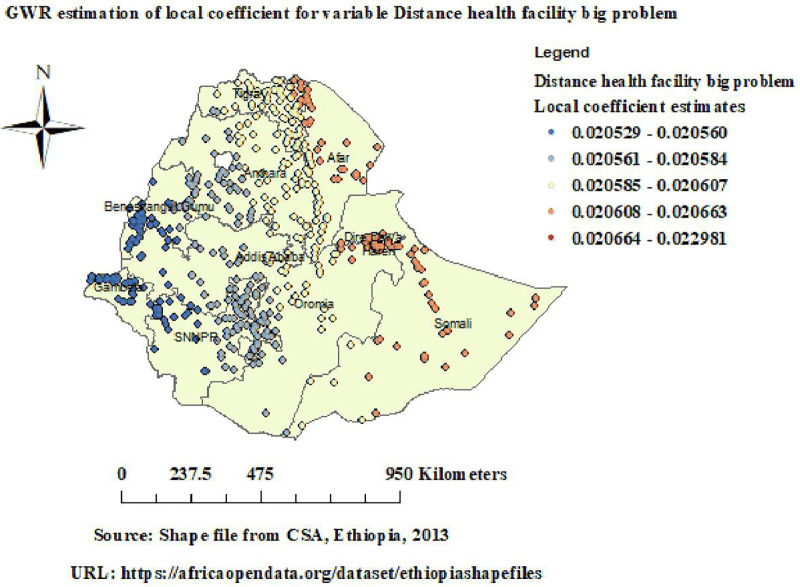
GWR of distance to health facility (big problem) in unintended pregnancy. Map data source: https://africaopendata.org/dataset/ethiopiashapefiles.

GWR analysis revealed that the relationship between distance to health facility (big problem) and unintended pregnancy varies across Ethiopia, with positive coefficients varying from (0.02) to (0.023). Strong positive coefficients varying from (0.021) to (0.023) and were observed in Eastern Tigray, Afar, Dire Dawa, and Somali (Fig 9).

## Discussion

Unintended pregnancies are a serious public health concern that has several effects on the health of mothers and children. Therefore, this study aimed to assess geospatial variation and factors of unintended pregnancy in Ethiopia using DHS 2016. Spatial autocorrelation revealed a statistically significant clustering pattern of unintended pregnancy. SaTScan analysis identified 171 primary spatial clusters (RR = 1.86, p-value less than 0.001). GWR revealed that maternal age (35–49 years), maternal primary education, high socioeconomic status, and distance to health facility (big problem) were statistically significant factors of unintended pregnancy in Ethiopia.

Spatial autocorrelation revealed a statistically significant clustering pattern of unintended pregnancy, with a Moran’s Index of 0.64, and a p-value less than 0.001 in Ethiopia. Hotspot analysis identified statistically significant hotspot areas of unintended pregnancy in Amhara, Addis Ababa, Oromia, and SNNPR; however, statistically significant coldspot areas of unintended pregnancy were observed in Afar, Dire Dawa, and Somali. Hotspot detection enables community-based initiatives to create awareness of unintended pregnancy, and this approach also guides healthcare infrastructure and strategic policy making to reduce inequities through evidence-based targeted interventions [30]. The highly predicted unintended pregnancy in Ethiopia was observed in Amhara, Addis Ababa, Oromia, and SNNPR. Spatial prediction enables health authorities to identify areas of high unintended pregnancy, which makes it possible to allocate resources and have a better understanding of spatial disparities and notifies policymakers about areas that require intervention [30]. Spatial SaTScan analysis identified a total of 171 statistically significant primary clusters, observed in Addis Ababa, Oromia, and SNNPR. In this window, the LLRs were 82.19, RR: 1.86, at a p-value of less than 0.001. It indicated that women inside the spatial window had an 86% higher risk of unintended pregnancy than women outside the window. These findings offer evidence and reveal the need for national health policy to target areas of high unintended pregnancy, so that public health resources can be allocated where they are most needed [30].

GWR analysis revealed that the relationship between maternal age (35–49 years) and unintended pregnancy varies across Ethiopia, with strong positive coefficients varying from (0.042) to (0.046) observed in Eastern Afar, Dire Dawa, and Somali. This suggests that as the proportion of women aged 35–49 years increases, the likelihood of unintended pregnancy also rises in these areas. A similar pattern was observed in a study in Uganda and Ethiopia, where unintended pregnancy was significantly higher among women aged 35 and above years compared to younger counterparts [33,34]. This could be because older women are less likely to take modern contraception and may not believe they are fertile, whereas younger women had less access to sexual and reproductive health information [35]. These findings highlight the necessity of focused reproductive health interventions for older women, particularly in areas where the rate of unintended pregnancies is high [36].

GWR analysis revealed that the relationship between maternal primary education and unintended pregnancy varies across Ethiopia, with strong positive coefficients varying from (0.031) to (0.034) observed in Eastern Afar, Dire Dawa, and Somali. This suggests that in areas where more women have primary-level education, the rate of unintended pregnancies tends to be higher. This result is inline with research done in Ethiopia [34,37]. This could be because unintended pregnancies are more common among women with only a primary level education, indicating that primary education may not be sufficient to provide enough understanding about sexual and reproductive health, which supports the idea that more educated women are more aware of their rights and have greater autonomy, control, and involvement in decisions regarding family planning and the use of contraception [34]. These findings emphasize the significance of ensuring secondary and higher education advancement for girls in addition to increasing their school enrollment, as this could greatly lessen the burden of unintended pregnancies by enhancing their knowledge, agency, and access to reproductive health services.

GWR analysis revealed that the relationship between high socioeconomic status and unintended pregnancy varies across Ethiopia, with strong positive coefficients varying from (0.017) to (0.019) observed in Eastern Tigray, Afar, Dire Dawa, and Somali. This implies that the rate of unintended pregnancies tends to raise in these areas as the percentage of women in the high socioeconomic status rises. These results both contradict and corroborate those of earlier research. While some suggest that women in poverty are more likely to give birth to an unintended child [38] others argue that high socioeconomic women were more likely to become pregnant or give birth to an unintended child [39]. In some contexts, women in the highest socioeconomic class may have higher rates of unintended pregnancy, possibly because they have desired family sizes, while in other settings, highest socioeconomic is protective [40]. This suggests that the relationship between wealth and unintended pregnancy is context-specific, varying by region, urban/rural location, and local sociocultural factors. These findings emphasize how crucial it is to adapt reproductive health initiatives to the socioeconomic circumstances of the local community.

GWR analysis revealed that the relationship between distance to health facility (a big problem) and unintended pregnancy varies across Ethiopia, with strong positive coefficients varying from (0.021) to (0.023) observed in Eastern Tigray, Afar, Dire Dawa, and Somali. This suggests that as the proportion of women with distance to a health facility (big problem) increased, unintended pregnancy also increased. This implies that unintended pregnancies are more common in women living farther away from a health facility. Limited physical access to reproductive health services is linked to lower contraceptive use and higher rates of unintended pregnancy, according to research done in Ethiopia and other low-resource settings [13,41,42]. This may be because women who live far from health facilities have transportation, financial, and logistical obstacles that prevent them from getting timely access to contraceptives, family planning counseling, and information on reproductive health [43].

### Strengths and limitations of study

The study employed DHS data collected by national standards to investigate the geospatial variation of unintended pregnancy. It employed spatial techniques to identify the problem areas. However, the study had limitations. First, 21 clusters with zero coordinates and 2 clusters with no matched values were excluded from the analysis. Since the DHS is cross-sectional, the nature of the data limits the ability to establish causality. To protect the privacy of the data, the location data values were moved 1–2 km for urban enumeration areas and 5 km for rural enumeration areas, and this may not give the actual case locations that there might be slight geographical variations from the real locations. The data were gathered in 2016 EDHS; while they offer insightful information about spatial patterns and related factors, the findings’ present applicability may be impacted by changes in reproductive health services, policy, and social norms over time. With observational research, there may be unmeasured confounders that could affect exposure variables and unintended pregnancy but were not included in the DHS dataset, such as partner-related characteristics, cultural norms, or the quality of healthcare facilities. Therefore, the results of this study were based on this limitation.

## Conclusions

The prevalence of unintended pregnancy in Ethiopia was high with significant variation across regions. Spatial autocorrelation revealed a statistically significant clustering pattern of unintended pregnancy. Hotspot analysis identified statistically significant hotspot areas of unintended pregnancy in Amhara, Addis Ababa, Oromia, and SNNPR. Statistically significant coldspot areas of unintended pregnancy were observed in Afar, Dire Dawa, and Somali. Spatial Kuldorff’s SaTScan identified 197 significant clusters of unintended pregnancy in Ethiopia. Significant factors of unintended pregnancy include maternal age (35–49 years), maternal primary education, high socioeconomic status, and distance to health facility. Therefore, policy and program decisions should be made carefully, even though these findings help direct focused initiatives and resource allocation.

## Abbrevation:

DHS: Demographic Health Survey
EDHS: Ethiopia Demographic Health Survey
GWR: Geographically Weighted Regression
OLS: Ordinary Least Square
